# Adolescents’ Assessment of Several Step Tracker Mobile Applications Based on Their Previous Level of Physical Activity

**DOI:** 10.3390/children12050554

**Published:** 2025-04-25

**Authors:** Nerea Gómez-Cuesta, Adrián Mateo-Orcajada, Lourdes Meroño, Lucía Abenza-Cano, Raquel Vaquero-Cristóbal

**Affiliations:** 1 Facultad de Deporte, UCAM Universidad Católica de Murcia, 30107 Murcia, Spain; ngomez8@ucam.edu (N.G.-C.); lmerono@ucam.edu (L.M.); labenza@ucam.edu (L.A.-C.); 2 Research Group Movement Sciences and Sport (MS&SPORT), Department of Physical Activity and Sport, Faculty of Sport Sciences, University of Murcia, 30720 Murcia, Spain; raquel.vaquero@um.es

**Keywords:** adolescents, dropout rate, mobile applications, mobile phone, problematic use

## Abstract

**Background:** Adolescents’ use of step tracker mobile applications (apps) could be influenced by their assessment of the mobile app used. **Objectives:** To analyze differences in the dropout rate, app assessment, and problematic mobile phone use based on prior physical activity levels and the app used. **Methods:** A study with a quasi-experimental design was carried out with the participation of 240 adolescents, who were further categorized as either active or inactive. The adolescents carried out a 10-week intervention promoted as a part of the physical education curriculum, three days a week, in which they randomly used the Pokémon Go, MapMyWalk, Pacer, or Strava mobile apps after school for cardiorespiratory training. **Results:** The results showed a higher dropout rate from Pokémon Go in the group of inactive adolescents (*p* = 0.012). No differences were found in the assessment of apps based on the level of activity of the adolescents, neither overall nor when analyzing this question based on the app used (*p* > 0.05). As for problematic mobile phone use, only previously inactive adolescents assigned to the Pacer group significantly increased their final score after the intervention (*p* = 0.044), with no changes being identified in the other groups or apps (*p* > 0.05). Furthermore, the active adolescents showed a positive relationship between the volume of training and problematic mobile phone use (*p* = 0.023), specifically with the conflicting use of this device (*p* = 0.017). **Conclusions:** The inactive adolescents had a higher dropout rate when using Pokémon Go. There were no differences in app assessment between the active and the inactive participants. However, the active adolescents showed a link between their training volume and problematic mobile phone use.

## 1. Introduction

Adolescence is a decisive stage for the establishment of healthy lifestyle habits [1] and for the prevention of chronic diseases [2,3]. Among the most important healthy habits, the practice of physical activity is considered fundamental for the maintenance of physical fitness [4], for the improvement of body composition [5], and for a healthier psychological state [6]. However, recent studies show that the abandonment of physical activity is increasing among adolescents, with up to 81% of adolescents not meeting the minimum physical activity recommendations established by the World Health Organization (WHO) [7].

To reverse this situation, interventions have been developed in the school and out-of-school contexts to promote the practice of physical activity, including active travel to school [8,9], active schools [10], active breaks [11], and out-of-school activities [12]. The results found in these studies have been encouraging, as they have shown increases in the participants’ levels of physical activity and improvements in their physical condition, as well as lower levels of adiposity, after the interventions [12,13]. However, despite the effectiveness shown in increasing the active time of the adolescent population [14], these programs have not been sufficiently stimulating for all adolescents, highlighting the lack of effectiveness in inactive adolescents [15]. Because of the above, inactive adolescents continued to show very low levels of physical activity after the interventions, which has a negative impact on their health [16].

Due to this, recent research has opted for the use of new technologies to promote physical activity in inactive and sedentary adolescent populations [17], as they provide sufficient novelty to generate adherence and obtain positive results [18]. These interventions have proven to be effective in reducing physical inactivity time, mainly in the adolescent population [17], but studies are still scarce, and their effectiveness seems moderate [17]. Among the most relevant interventions involving technological devices with adolescents, step tracker mobile apps stand out, as they have proven to be useful tools for the promotion of physical activity among adolescents [19]. These interventions are characterized by an increase in participants’ daily steps and are promoted by the physical education curriculum to encourage after school physical activity [19]. However, this type of intervention has a major handicap, in that the initial novelty wears off after the first few weeks of the intervention [20], which considerably reduces adherence and effectiveness. In this regard, previous research conducted on adolescents has shown high dropout rates during the intervention, with differences between the mobile applications used (Strava 33%, Pacer 41%, MapMyWalk 45%, and Pokémon Go 41%) [21]. Furthermore, no differences were found in the dropout rate according to factors such as the weight status of the adolescents, being similar in normal-weight and overweight/obese adolescents [22]. Therefore, dropout seems to be a general problem in the use of mobile apps by adolescents, regardless of their characteristics.

Little is known about the reasons for adolescents’ loss of adherence to this type of intervention over time, but recent research has shown the importance of individualization in physical activity interventions with technological devices [17]. In this regard, the mobile physical activity apps used in previous research [19], although effective, lacked individualization for this population. This is because, although they rate most of the features of these mobile apps positively, with moderate and high scores for engagement, functionality, aesthetics, and informativeness, the score given for usability and perceived impact by both normal-weight and overweight adolescents is low, mainly for apps such as Pokémon Go [21,22]. Furthermore, this assessment could be affected by the distance covered with the application, since it would imply a longer time spent using the application [21]. Therefore, the longer the distances traveled by the adolescents while using the apps, the more the observed limitations of these mobile applications in terms of their functionality and design, as well as in the information they provide and the necessary requirements of the smartphones for them to work, point out that they are not designed to be used by adolescents [23].

On the other hand, it is unknown whether the increased use of mobile devices could have detrimental effects on adolescent health, as previous research has shown that these interventions can increase the time spent using other non-research related mobile apps [24]. This does not seem to be the case for physical activity mobile apps, as no increases in problematic mobile phone use were observed after the intervention in neither normal-weight nor overweight adolescents [22]. However, it has been observed that the longer amount of time spent using these applications, due to the greater distances traveled, is related to greater problematic use of the mobile phone [22], so more studies are needed to discover whether the increased use of step tracker applications truly has an effect on the problematic use of mobile phones.

However, no research was found that analyzed the assessment by adolescents of the physical activity mobile applications used based on the previous level of physical activity. Therefore, this is a largely unexplored area, but of great relevance for the future effectiveness of mobile-app-based interventions. In this regard, inactive adolescents, who have lower baseline physical activity levels, showed superior improvements in their physical activity levels as compared to active adolescents after follow-up interventions with mobile apps [25].

In view of the existing scientific gap in this field of knowledge, the objectives of the present research were as follows: (a) to analyze the differences in the dropout rate of an intervention based on the use of step tracker mobile apps during outside-of-school hours promoted by the physical education curriculum, as a function of the previous activity level of the adolescents and the mobile application used; (b) to establish the differences in the assessment of mobile applications by active and inactive adolescents; (c) to determine whether the use of the step tracker mobile apps produces changes in problematic mobile phone use; and (d) to analyze whether the distance traveled with the use of the step tracker mobile apps is related to the assessment of the mobile application and to the problematic use of this device.

Based on the stated objectives, the research hypotheses are as follows: there will be differences in the dropout rate after the use of step tracker mobile apps depending on the previous level of physical activity of the adolescents and the mobile app used (H1); there will be differences in the assessment of mobile apps by active and inactive adolescents (H2); there will be an increase in problematic mobile phone use after the intervention with step tracker mobile apps, regardless of the previous level of physical activity and the app used (H3); and the distance traveled while using step tracker mobile apps will be related to the assessment of the mobile application and to the problematic use of this device (H4).

## 2. Materials and Methods

### 2.1. Design

A ten-week study with a quasi-experimental design was conducted with two experimental groups (active and inactive adolescents), who were subsequently randomly assigned to one of the four selected mobile apps (Strava, Pokémon Go, Pacer, and MapMyWalk). The study variables (pre- and post-test) were measured before and after the intervention. The use of the mobile application during after-school hours was promoted by the physical education curriculum. Non-probabilistic convenience sampling was used.

The study was approved by the institutional ethics committee of the Catholic University of Murcia (code: CE022102) and followed the principles of the Declaration of Helsinki. The study followed the Consolidated Standards of Reporting Trials (CONSORT) guidelines [26], and the measurement protocol was registered prior to the start of the intervention at ClinicalTrials.gov (code: NCT04860128).

The sample size was calculated according to the standard deviation (SD) of previous studies that had used step tracker mobile apps to increase physical activity in adolescent populations (SD = 0.67) [19]. Thus, the minimum sample size required for the study was 100 adolescents per experimental group, for a confidence interval (CI) of 95% and an estimated error (d) of 0.13 for the physical activity level variable. The statistical program used to calculate the sample size was Rstudio 3.15.0 (Rstudio Inc., Boston, MA, USA).

### 2.2. Participants

Two Compulsory Secondary Education (ESO) centers were selected for this research. The schools were chosen because they had the largest number of adolescents enrolled in ESO. After the selection of the centers, the members of the management team were informed of the research objectives and procedures. Once approval was received, meetings were held with the teachers responsible for the physical education curriculum and, subsequently, with the students and their parents. Participation was completely voluntary. The data collection and the subsequent publication of the anonymous data were authorized by the informed consent signed by the adolescents and their parents. The inclusion criteria were as follows: (a) aged between twelve and sixteen years old; (b) attending ESO. The exclusion criteria were as follows: (a) not having their own mobile phone; (b) not attending the post-test measurements.

**Figure 1:**
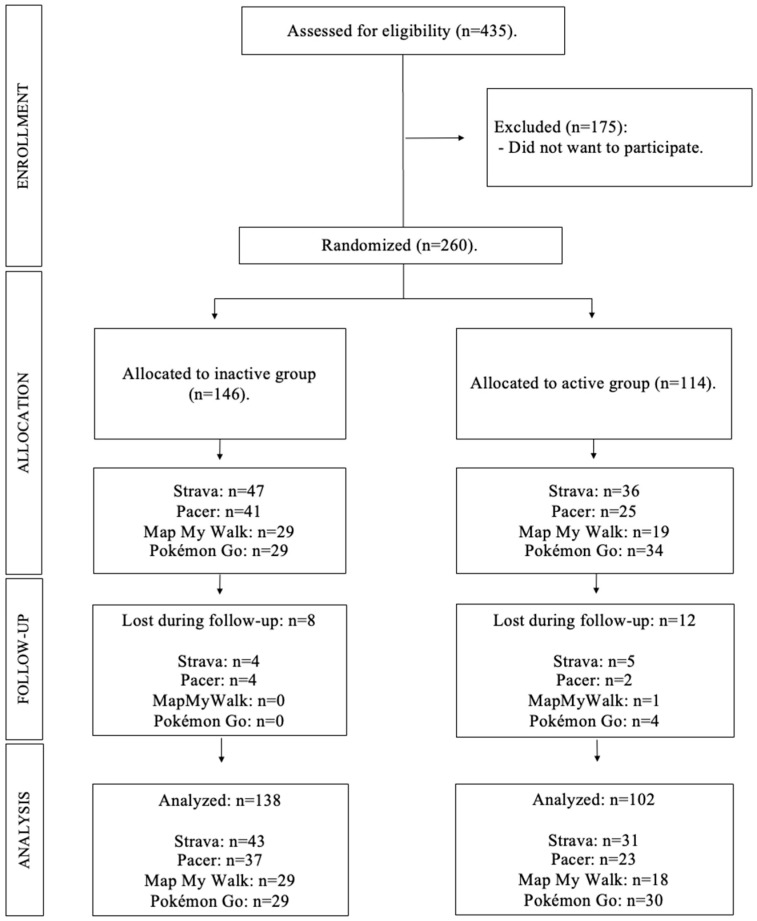
Sample flowchart.

The sample flow diagram is shown in Figure 1. In total, 435 adolescents were potentially eligible for the research study. The initial sample consisted of 260 adolescents, with 240 adolescents of between twelve and sixteen years of age (mean age: 13.76 ± 1.41 years) ultimately participating in the intervention. The participation of active and inactive adolescents was similar at the beginning of the study (146 active and 114 inactive), as well as that of males and females (135 males and 125 females).

### 2.3. Randomization and Blinding

The principal investigator, together with other investigators not involved in the study, carried out the randomization process using a computer-generated random number table. Adolescents belonging to the same class were randomly assigned to one of four mobile apps (Strava, Pacer, MapMyWalk, and Pokémon Go). The small differences between the active and inactive adolescents in each of the classes explain the differences in the number of adolescents assigned to each application. Baseline measurements were performed after the randomization process. The researchers remained blinded as to the application used by each adolescent and as to whether he or she was active or inactive.

### 2.4. Instruments

The “Physical Activity Questionnaire for Adolescents” questionnaire (PAQ-A) [27] was used to establish the level of physical activity of the adolescents. This questionnaire was previously validated in Spanish for the adolescent population, and an intraclass correlation coefficient of 0.71 was calculated for the final score of the questionnaire [28]. It is composed of 9 items, the first 8 of which are answered with a Likert scale of 1 to 5 points (1: a low level of physical activity; 5: a high level of physical activity), while the last item is answered with a dichotomous question (yes or no) to discover the physical activity performed by the subject the week prior to completing the questionnaire. This questionnaire provides information on the physical activity status of adolescents by means of the arithmetic mean of the first eight items. This score allows a cut-off point to be established to classify adolescents as either active (≥2.75) or inactive (<2.75) [29].

To assess problematic mobile phone use, the “Questionnaire of Mobile-Phone-Related Experiences” (CERM) [30] was used. This questionnaire is composed of 10 items that are answered with a Likert scale of 1 to 4 points (1: never; 4: almost always), with the final score being the sum of the 10 items. Higher scores are related to more problematic mobile phone use. This questionnaire provides two dimensions, each consisting of five items: conflicting use and emotional use. With these items, the questionnaire measures the following: (a) the problematic use of the mobile phone, with a score of above 24; (b) occasional problems, with a score ranging from 16 to 23 points; and (c) the absence of problems, with a score of below 16. This questionnaire was previously validated in the adolescent population and shows a high internal consistency for the questionnaire in general (α = 0.80) and for both dimensions (conflictive use: α = 0.81; emotional use: α = 0.75) [30].

The assessment of the applications was carried out with the “User version mobile application rating scale” (uMARS) [31]. This instrument is composed of 26 items to assess the objective and subjective quality of mobile applications. It is completed with a Likert scale from 1 to 5 points (1: inadequate; 5: excellent). The objective quality is divided into four sections: engagement (internal consistencies: α = 0.80), functionality (internal consistencies: α = 0.70), aesthetics (internal consistencies: α = 0.71), and information (internal consistencies: α = 0.78) [31]. The engagement section assesses the entertainment, interest, personalization, interaction, and target audience of the application; the functionality section rates the performance, ease of use, application navigation, and gesture design of the application; the aesthetics section assesses the layout, graphics, and visual appearance of the application; and the information section analyzes the quality, quantity, and credibility of the information provided [31]. The maximum score for each category is five points for engagement (items 1–5), five points for functionality (items 6–9), five points for aesthetics (items 10–12), and six points for information (items 13–16). The average of these four dimensions is the total score for the objective quality. The subjective part includes ten questions that assess usability and perceived impact. The internal consistency of the scale in the original version was excellent (α = 0.90) [31], and it remains so in the Spanish version (α = 0.89) [32].

### 2.5. Procedure

Prior to the start of the intervention (pre-test), the adolescents completed the PAQ-A and CERM questionnaires. For this purpose, a classroom was used in which the adolescents remained quiet and there were no external factors that could distract them or hinder their understanding of the questionnaires. This was followed by the step tracker mobile app intervention, which lasted ten weeks, during which the adolescents had to use the mobile app a minimum of three times a week. Before starting, each class was given an explanation about how the application works, and any doubts were answered. A researcher oversaw the recording of the distance completed by each adolescent on a weekly basis. At the end of the intervention (post-test), the adolescents completed the CERM questionnaire and the uMARS questionnaire in the same environmental conditions as in the pre-test. The researchers did not influence the adolescents’ answers and only resolved possible doubts.

### 2.6. Mobile Apps Interventions

First, the correct functioning of the application was explained to the adolescents, as well as the way in which they were to record the distance covered in the application. Subsequently, the 10-week intervention began, in which the adolescents used the corresponding mobile application three times a week after school hours. These applications were selected for their similarity to each other, as well as for including numerous behavioral change techniques [33]. Strava, Pacer, and MapMyWalk are considered pedometers that include messages and reminders to encourage physical activity; Pokémon Go is a video game in which teens find different Pokémon to capture as they walk and receive rewards for the distance covered [34,35].

During the ten weeks of the intervention, the distance that the adolescents had to walk each week was progressively increased. The first week started with a daily distance of 4.5 km, which corresponds to about 7152 steps [36]; the distance was set according to Morency et al. [37], who suggested that 1 km for this age range corresponds to approximately 1565 steps. This distance was increased by 600 steps per week until reaching a distance of 12,520 steps in the tenth week, for each day that the mobile application was used, which corresponds to 8 km, the minimum distance recommended to reach a healthy physical activity habit [36]. The target distance for each session could be completed by the adolescents in a single session or in several sessions during after-school hours. Each week, the same researcher was in charge of recording the distance completed by each adolescent in each of the sessions carried out. In addition, he also noted whether the daily distance was completed in one or several sessions. The records of activities corresponding to school hours were excluded from the final analysis. Before starting, the adolescents were informed that completing the intervention would increase their final grade by 10% in the physical education course. Those adolescents who did not travel the full distance planned for the intervention were not excluded from the intervention and continued to be part of the study sample (n).

### 2.7. Data Analysis

The normality of the data was assessed with the Kolmogorov–Smirnov test, as well as with analyses of skewness and kurtosis. Since the variables followed a normal distribution, parametric tests were used for their analysis. A paired-samples Student’s *t*-test was performed to determine the differences in the levels of physical activity of the active and the inactive adolescents between the pre- and post-test. The Chi-square test (χ^2^) was used to analyze the differences between the active and the inactive group in the days they walked and ran in the pre- and post-test; as well as to analyze the differences in adherence and dropout rates as a function of the level of physical activity of the adolescents. The corrected standardized residuals were used to determine significance, establishing ± 1.96 as the reference value. The contingency coefficient was used in the 2 × n tables to obtain the statistical value. The maximum expected value was 0.707; r < 0.3 indicated a low association; r < 0.5 indicated a moderate association; and r > 0.5 indicated a high association [38]. Two ANOVAs were carried out, the first to establish the differences in the value of the applications between the active and the inactive adolescents, and the second to establish the differences in the assessment of the different mobile applications among adolescents of the same level of physical activity. A Bonferroni post-hoc analysis was used to determine between which mobile apps the differences were found. A multivariate ANOVA (MANCOVA) was performed to determine the differences in problematic mobile phone use according to the level of physical activity and the mobile application used, considering the covariate quality of the app. Finally, a Pearson correlation analysis was performed to establish the relationship between the distance traveled with the use of the app, the assessment made, and the problematic use of the mobile phone. The partial eta squared (η^2^) was used to calculate the effect size, and it was defined as small if the ES ≥ 0.10; moderate if the ES ≥ 0.30; large if the ES ≥ 1.2; or very large if the ES ≥ 2.0, with an error of *p* < 0.05 [39]. A value of *p* < 0.05 was set to determine statistical significance. The statistical analysis was performed using the SPSS statistical package (v.25.0; SPSS Inc., Chicago, IL, USA).

## 3. Results

After the intervention with mobile applications, an increase in the level of physical activity was observed in the group of inactive adolescents (*p* < 0.001; mean diff pre-post: −0.31) but not in the group of active adolescents (*p* = 0.582; mean diff pre-post: 0.02). However, Table 1, in which the differences between the walking and running days of the active and the inactive adolescents in the pre- and post-tests are reported, shows that the differences existing in the pre-test walking days between the active and the inactive groups cease to be significant in the post-test because there is an increase in walking days in the inactive adolescents (2–3 days a week mainly), but also in the active ones, increasing from 17. 6% to 31.4% for the adolescents who walked three days a week and decreasing from 11.8% to 5.9% for those who only walked one day a week. As for running, the differences found in the pre-test were maintained in the post-test.

The dropout rate according to the level of physical activity and the mobile app used are shown in Table 2. No significant differences were found according to the different mobile applications in the total sample of adolescents (*p* = 0.318). However, significant differences were found between the applications in the group of inactive adolescents (*p* = 0.012), with the dropout rate being 44.8% for Pokémon Go. No differences were found in the active group (*p* = 0.176).

Table 3 shows the differences in the assessment of mobile applications between the active and the inactive adolescents. The results showed no differences between the two groups in engagement (*p* = 0.560–0.761), functionality (*p* = 0.566–0.977), aesthetics (*p* = 0.669–0.960), information (*p* = 0.408–0.862), usability (*p* = 0.299–0.805), or perceived impact (*p* = 0.195–0.934) for any of the apps analyzed.

Table 4 shows the differences in the assessment of mobile apps in active and inactive adolescents according to the mobile application used. No significant differences were found in any of the variables analyzed in the group of inactive adolescents (engagement: *p* = 0.675; functionality: *p* = 0.223; aesthetics: *p* = 0.345; information: *p* = 0.304; usability: *p* = 0.335; perceived impact: *p* = 0.599), nor in that of the active adolescents (engagement: *p* = 0.972; functionality: *p* = 0.477; aesthetics: *p* = 0.622; information: *p* = 0.255; usability: *p* = 0.163; perceived impact: *p* = 0.484).

The differences between the pre- and post-tests regarding problematic mobile phone use in the active and the inactive adolescents as a function of the mobile application used are shown in Table 5. Significant differences in the final CERM questionnaire score were found only in the inactive adolescents who used the Pacer app, with an increase in the score after the intervention (*p* = 0.044). The inclusion of the covariate “quality of the app” was not shown to significantly influence the final score of the questionnaire (*p* = 0.070–0.955), neither in the dimension of conflicting *(p* = 0.082–0.738) nor emotional (*p* = 0.104–0.919) use in any of the groups.

Table 6 shows the correlation analysis of the training volume, app rating, and the overall level of problematic mobile phone use in the active and the inactive adolescents. In the group of inactive adolescents, there was no significant correlation between the volume of training completed and the rest of the variables analyzed (*p* = 0.168–0.992), while in the group of active adolescents, a positive relationship was found between the training volume and the problematic use of mobile phones (*p* = 0.023), specifically with the conflicting use of this device (*p* = 0.017).

## 4. Discussion

The first objective of this research was to analyze the differences in the dropout rate of an intervention based on the use of step tracker mobile apps during outside-of-school hours promoted by the physical education curriculum according to the previous level of physical activity of the adolescents and the mobile application used. The results of the present study show that the intervention with mobile applications increased the level of physical activity after the intervention, mainly in the inactive group, but also produced an improvement in the walking pattern of both the active and the inactive groups, increasing the weekly frequency at the end of the intervention. Therefore, this type of intervention to promote the completion of steps could be effective to increase physical activity and walking, regardless of the initial characteristics of the population [40,41]. Among the apps analyzed, Pokémon Go had the highest abandonment rate among the inactive adolescents. These results are similar to previous research, in which it was found that a high percentage of teens stopped using the Pokémon Go mobile app after an eight-week intervention program [42]. One possible explanation for these results is that despite the app being considered an appropriate tool to promote physical activity in adolescents [35], the novelty effect of the app wears off after the first few weeks, so that adolescents may stop using it as they find the content of the game repetitive, as has been found in previous research with adults [43]. Another explanation could be that adolescents find problems related to the recording of the distance traveled in the Pokémon Go application [44] or with the risk of running out of battery during the activity, since the geolocation function of this application consumes a high percentage of the mobile device’s battery [45]. The relevance of the results lies in the fact that the design of mobile applications should be more responsive to the needs and requirements of the inactive adolescent population, as they do not have an established walking habit, and the type of mobile application could determine the success of interventions aimed at increasing physical activity.

This fact is important, as previous research that analyzed the influence of other conditions, such as participants’ weight status, on the abandonment of interventions with mobile physical activity applications did not find significant differences [22]. These results are in line with those obtained by the group of active adolescents, where no significant differences were found with respect to the abandonment rate among the different mobile applications. One possible explanation for these results could be that the intervention included a reward as a part of the final grade of the physical education course, so it is likely that the active adolescents, who tend to obtain better grades in this subject [46,47], completed the subject regardless of the mobile application used [46,47]. On the other hand, active adolescents usually present an intrinsic motivation to move and perform physical activity [48,49], which does not usually occur in inactive adolescents who require extrinsic motivation, such as the presence of attractive elements such as gamification [48,50], and this could be the reason why there were differences between the dropout rates of the inactive adolescents depending on the mobile app. However, future research is needed to continue analyzing the reasons, as well as the conditions, that may influence the abandonment of mobile app interventions.

Based on the results obtained in the present investigation, the first research hypothesis (H1), which indicated that there would be differences in the dropout rate after the use of step tracker mobile apps depending on the previous level of physical activity of adolescents and the mobile application used, can be partially accepted. This is because significant differences were only found in the dropout rate of the inactive adolescents, with the Pokémon Go mobile app having the highest dropout rate, although there were no significant differences among active adolescents; nor among the rest of the applications in the inactive category.

The second objective of the present research was to establish the differences between the assessments made of the mobile applications by the active and the inactive adolescents. The results showed no significant differences between the active and the inactive adolescents in any of the dimensions assessed for any of the mobile applications used, with high scores observed in all cases. These results are similar to those from previous research with adults, in which app assessment was positive [51]. Also, they are similar to those from previous studies, in which the scores of the apps included in the intervention for use in ESO physical education classes were high [52]. A possible explanation for these results could be that the mobile applications used in the present research were selected because they included a high number of behavioral change techniques, which could have favored the assessment of these applications by adolescents [33,53]. Previous research has shown that the use of applications that incorporate behavioral change techniques allow the user to set goals based on their physical activity needs, as well as to obtain feedback on their performance and knowledge about exercise execution [54]. Given that feedback, social support, reminders, rewards, and self-monitoring are the most relevant behavioral change techniques for adolescents in physical activity mobile applications [55], they could positively affect adolescents’ motivation and commitment to physical activity [55,56], facilitating a better assessment of these mobile applications. This, considering the results of the present investigation, seems to be independent of the initial level of physical activity of the participants. Along the same lines, a recent study found similar results in another hard-to-reach population, namely overweight and obese adolescents, finding that they rate step tracker applications similarly to normal-weight participants [22].

Based on the results obtained, the second research hypothesis (H2), which indicated that the assessments of mobile applications by the active and the inactive adolescents would be different, can be rejected. This is because no significant differences were found in any of the rated dimensions of the mobile applications between the active and the inactive users.

The third objective of the present research was to determine whether the use of step tracker mobile apps produced modifications in adolescents’ problematic mobile phone use. In this regard, the results obtained showed a significant increase in problematic mobile phone use among inactive adolescents who used the Pacer application. These results differ from those of previous research, in which no differences were found in problematic mobile phone use by normal-weight or overweight adolescents following the use of this type of mobile physical activity application [22]. A possible explanation for these results could be based on the fact that previous research has shown that mobile applications used as pedometers have complex interfaces that make it difficult to track interventions [21]. This is the case of Pacer, in which, moreover, it seems that the information included is not sufficiently attractive to teenagers as compared to that included in other applications with similar characteristics [21]. This could be the reason why the inactive adolescents, for whom the interest in physical activity is lower as compared to the active adolescents [57], spent more time using the mobile device for purposes other than the use of mobile physical activity applications. The problem of the use of mobile applications for purposes other than those proposed in the intervention had already been observed in previous research [58,59], but the results of the present study show that inactive adolescents appear to be more vulnerable and more likely to misuse these devices.

However, the present research shows that the quality of the application as perceived by the adolescents was not a determinant factor on the increase of problematic mobile phone use, which is in line with previous research, in which an objective assessment of the mobile application did not significantly influence problematic mobile phone use in either normal-weight or overweight adolescents [22]. These results are relevant, because, regardless of the adolescents’ perception of this type of mobile app, their problematic use of the mobile phone was not affected. This could be due to the fact that some of the previous research in which an increase in problematic mobile phone use was observed was carried out in the school environment [60]. In that setting, adolescents tend to use mobile applications other than the one requested, so the use of the mobile device is prohibited, but as a rebellious response, more than 60% of them made use of the mobile device for non-educational purposes during school hours [61,62]. However, by promoting the use of mobile physical activity apps in out-of-school settings, it is possible that the time that was not spent using the app was also not spent using the mobile device for other problematic purposes, since the leisure possibilities are greater as compared to those in the school setting [63,64]. However, future research is needed to analyze this issue and establish a more rigorous analysis of the time spent using the mobile phone.

The results allow us to partially accept the third research hypothesis (H3), which indicated that there would be an increase in problematic mobile phone use after the intervention with step tracker mobile apps, regardless of the previous level of physical activity and the application used. This is because significant differences in problematic mobile phone use were found only among the inactive adolescents who used the Pacer application.

The fourth objective of the present investigation was to analyze whether the distance traveled with the use of the step tracker mobile apps was related to the assessment of the mobile application and to the problematic use of this device. The results obtained showed no significant relationships between any of the variables analyzed in the group of inactive adolescents, while in the group of active adolescents, a positive relationship was found between the volume of training and the problematic use of mobile phones. This seems to indicate that active adolescents who travel a greater training distance use mobile devices to a greater extent and more problematically. It is likely that active adolescents, having a higher level of physical activity, traveled a greater distance each time they used the mobile application [65], which would lead to more training time and the possibility of using this device for other purposes. These results are in line with previous studies, in which it was observed that normal-weight adolescents who traveled a greater distance using mobile applications had a more problematic use of mobile devices [22]. Therefore, the findings of the present investigation make a relevant contribution to this body of knowledge, since they show that depending on the previous level of physical activity, there may be differences in the problematic use of mobile devices when a longer training distance is covered.

With the results obtained, the fourth research hypothesis (H4) can be partially accepted, as it indicated that the distance traveled with the use of the step tracker mobile apps would be related to the assessment of the mobile application and to the problematic use of this device. This is because significant differences were found in conflicting mobile phone use in active adolescents, but no significant differences were observed in inactive adolescents or in the assessment made of mobile applications in either active or inactive adolescents.

Regarding the practical applications of the present research, step tracker mobile apps are considered a decisive tool with which to increase physical activity levels and improve health parameters among the adolescent population, which can be promoted by the curriculum of physical education. Making use of these applications as tools within this curriculum could be a favorable strategy to promote the practice of physical activity in the outside-of-school context. In addition, the use of these mobile applications in the out-of-school environment also has benefits in the school environment. Thus, it has been observed that the use of these devices can increase motivation and engagement in physical activity, increasing participation in physical education classes [66,67]. On the other hand, these mobile applications provide the possibility to better understand the concepts related to physical education, facilitating the learning of the concepts worked on in class [68,69]. Moreover, these devices can be used by teachers as a complement to the subject, as they provide feedback and can be used to propose physical education homework that is done outside the school environment but has a direct impact on the grade obtained in the classroom [66,68]. Applications that include behavioral change techniques are proving to be effective tools for immediately providing adolescents with meaningful information about their health status. More specifically, in light of the results of the present research, the Strava and MapMyWalk applications could be the most effective for promoting the completion of steps in the after-school schedule, as they were the ones that showed the lowest dropout rate and the ones that did not have negative consequences with their use.

The present research is not without limitations. First, the PAQ-A questionnaire used to assess the activity level of the study population is a subjective measurement that records the physical activity performed in the previous week, which could have influenced the results of the investigation. The problematic use of the mobile phone was measured with an instrument that, although valid and reliable, makes a subjective assessment of this variable, so it would be advisable to include an objective assessment of the time spent using the mobile application. On the other hand, for the quality of the applications studied, the uMars scale was used, which could include terms that are too complex for the adolescent population, which may have influenced the results of the research. Due to the sample included in each application, it was not possible to perform the analysis according to gender, which would have been interesting due to the differences in the practice of physical activity between adolescent males and females [70]. Although the ACSM indicates that it is the total cumulative training volume that is important [71], the fact that some adolescents completed the proposed distance for each day in a single session may have produced different effects compared to those who completed this distance in several sessions per day, so future research is needed to analyze the differences according to the method of reaching the total set volume. Finally, not having a record of the steps taken by the adolescents before starting the intervention or at the end of the intervention is an important limitation when conducting research with mobile applications to promote walking, so it is an aspect of great relevance to consider in future research.

## 5. Conclusions

It can be concluded that there are differences in the dropout rates of participants using step tracker mobile apps promoted by the curriculum of physical education for use after school hours depending on their level of physical activity, with the inactive adolescents who used Pokémon Go having the highest dropout rate in this investigation. On the other hand, it should be noted that although there were no differences between the active and the inactive adolescents in the assessment of mobile applications, there were differences in the problematic use of the mobile phone, where the inactive adolescents who used Pacer showed a significant increase in this variable. Finally, special attention should be paid to active adolescents who travel a greater distance using mobile apps as this increase in the time required to complete a longer workout also appears to increase problematic mobile device use.

## Figures and Tables

**Table 1:** The analysis of the evolution of walking and running days in the active and the inactive adolescents.

Time Moment	Activity Level	Weekly Activity Days					Adj Res. Dropout/Non Dropout	Group Diff. (χ^2^, *p*)	Contingency Coefficient
		0/1	2	3	4	5			
Walking									
Pre	Active	12 (11.8%)	29 (28.4%)	18 (17.6%)	17 (16.7%)	26 (25.5%)	−2.6; 2.6	12.16; *p* = 0.016	0.220
	Inactive	31 (22.6%)	45 (32.8%)	30 (21.9%)	8 (10.2%)	17 (12.4%)			
Post	Active	6 (5.9%)	28 (27.5%)	32 (31.4%)	15 (14.7%)	21 (20.6%)	−1.2; 1.2	4.30; *p* = 0.367	0.134
	Inactive	13 (9.5%)	48 (35.0%)	42 (30.7%)	13 (9.5%)	21 (15.3%)			
Running									
Pre	Active	13 (12.7%)	31 (30.4%)	25 (24.5%)	22 (21.6%)	11 (10.8%)	5.4; −5.4	47.47; *p* < 0.001	0.446
	Inactive	62 (45.3%)	47 (34.3%)	20 (14.6%)	4 (2.9%)	4 (2.9%)			
Post	Active	14 (13.7%)	38 (37.3%)	22 (21.6%)	19 (18.6%)	9 (8.8%)	3.7; −3.7	25.55; *p* < 0.001	0.327
	Inactive	48 (35.0%)	42 (30.7%)	36 (26.3%)	6 (4.4%)	5 (3.6%)			

**Table 2:** Dropout rate according to the previous level of physical activity and the mobile app used.

App Used	Initial Sample (n)	Final Sample (n, %)	Dropout (n; %)	Adj Res. Dropout/Non Dropout	Group Diff. (χ^2^, *p*)	Contingency Coefficient
Total sample according to mobile app used						
Pokémon Go	59	42 (71.19%)	17 (28.81%)	−1.7/1.7	χ^2^ = 3.525; *p* = 0.318	0.120
Strava	74	59 (79.73%)	15 (20.27%)	0.1/−0.1		
Pacer	60	49 (81.67%)	11 (18.33%)	0.6/−0.6		
MapMyWalk	47	40 (85.11%)	7 (14.89%)	1.1/−1.1		
Sample divided according to level of physical activity and app used						
Pokémon Go—IN	29	16 (55.2%)	13 (44.8%)	−3.2/3.2	χ^2^ = 10.997; *p* = 0.012	0.272
Strava—IN	43	37 (86.0%)	6 (14.0%)	1.6/−1.6		
Pacer—IN	37	31 (83.8%)	6 (16.2%)	1.1/−1.1		
MapMyWalk—IN	29	23 (79.3%)	6 (20.7%)	0.3/−0.3		
Pokémon Go—A	30	26 (86.7%)	4 (13.3%)	0.9/−0.9	χ^2^ = 4.945; *p* = 0.176	0.215
Strava—A	31	22 (71.0%)	9 (29.0%)	−1.8/1.8		
Pacer—A	23	18 (78.3%)	5 (21.7%)	−0.4/0.4		
MapMyWalk—A	18	17 (94.4%)	1 (5.6%)	1.6/−1.6		

**Table 3:** Differences in mobile app assessment between active and inactive adolescents.

	App Used	Active	Inactive	Mean Diff. (Normal-Over)	F	*p*	95% CI	Effect Size
Engagement	Pokémon Go	3.43 ± 1.39	3.34 ± 1.49	0.095 ± 0.320	0.089	0.766	−0.535; 0.726	0.001
	Strava	3.52 ± 1.16	3.43 ± 1.11	0.088 ± 0.289	0.093	0.761	−0.482; 0.659	0.001
	Pacer	3.38 ± 1.14	3.51 ± 0.80	−0.131 ± 0.326	0.161	0.689	−0.774; 0.512	0.001
	Map My Walk	3.37 ± 1.36	3.15 ± 1.42	0.215 ± 0.369	0.340	0.560	−0.511; 0.941	0.001
Functionality	Pokémon Go	3.39 ± 1.40	3.28 ± 1.56	0.107 ± 0.338	0.101	0.751	−0.558; 0.773	0.001
	Strava	3.68 ± 1.23	3.67 ± 1.15	0.009 ± 0.306	0.001	0.977	−0.593; 0.611	0.001
	Pacer	3.96 ± 1.23	3.92 ± 0.80	0.038 ± 0.344	0.012	0.913	−0.641; 0.716	0.001
	Map My Walk	3.68 ± 1.49	3.46 ± 1.58	0.224 ± 0.389	0.330	0.566	−0.543; 0.990	0.001
Aesthetics	Pokémon Go	3.39 ± 1.46	3.24 ± 1.71	0.148 ± 0.344	0.184	0.669	−0.530; 0.825	0.001
	Strava	3.67 ± 1.23	3.68 ± 1.18	−0.016 ± 0.311	0.002	0.960	−0.629; 0.598	0.001
	Pacer	3.86 ± 1.15	3.76 ± 0.88	0.098 ± 0.351	0.079	0.780	−0.593; 0.790	0.001
	Map My Walk	3.52 ± 1.51	3.39 ± 1.48	0.128 ± 0.396	0.104	0.748	−0.653; 0.909	0.001
Information	Pokémon Go	3.21 ± 1.50	3.15 ± 1.72	0.062 ± 0.354	0.030	0.862	−0.635; 0.759	0.001
	Strava	3.79 ± 1.16	3.57 ± 1.24	0.221 ± 0.320	0.475	0.492	−0.410; 0.851	0.002
	Pacer	3.87 ± 1.25	3.76 ± 0.94	0.113 ± 0.361	0.098	0.755	−0.598; 0.824	0.001
	Map My Walk	3.71 ± 1.55	3.37 ± 1.57	0.338 ± 0.408	0.686	0.408	−0.466; 1.141	0.003
Usability	Pokémon Go	2.62 ± 1.37	2.54 ± 1.43	0.074 ± 0.297	0.061	0.805	−0.512; 0.659	0.001
	Strava	3.17 ± 1.00	2.89 ± 1.01	0.280 ± 0.269	1.084	0.299	−0.250; 0.809	0.005
	Pacer	3.25 ± 1.03	3.03 ± 0.72	0.216 ± 0.303	0.510	0.476	−0.380; 0.813	0.002
	Map My Walk	3.00 ± 1.31	2.72 ± 1.28	0.284 ± 0.342	0.691	0.407	−0.390; 0.959	0.003
Perceived impact	Pokémon Go	2.81 ± 1.52	2.66 ± 1.56	0.156 ± 0.336	0.215	0.643	−0.507; 0.818	0.001
	Strava	3.17 ± 1.23	2.77 ± 1.14	0.395 ± 0.304	1.689	0.195	−0.204; 0.995	0.007
	Pacer	3.09 ± 1.26	3.06 ± 0.90	0.028 ± 0.343	0.007	0.934	−0.647; 0.704	0.001
	Map My Walk	2.65 ±1.40	2.75 ± 1.38	−0.105 ± 0.387	0.073	0.787	−0.868; 0.659	0.001

**Table 4:** Differences in the mobile app assessments by the active and the inactive adolescents according to the app used.

	Physical Activity Level	Pokémon Go	Strava	Pacer	MapMyWalk	F	*p*	Effect Size
Engagement	Active	3.43 ± 1.39	3.52 ± 1.16	3.38 ± 1.14	3.37 ± 1.36	0.078	0.972	0.001
	Inactive	3.34 ± 1.49	3.43 ± 1.11	3.51 ± 0.80	3.15 ± 1.42	0.511	0.675	0.007
Functionality	Active	3.39 ± 1.40	3.68 ± 1.23	3.96 ± 1.23	3.68 ± 1.49	0.832	0.477	0.011
	Inactive	3.28 ± 1.56	3.67 ± 1.15	3.92 ± 0.80	3.46 ± 1.58	1.473	0.223	0.019
Aesthetics	Active	3.39 ± 1.46	3.67 ± 1.23	3.86 ± 1.15	3.52 ± 1.51	0.590	0.622	0.008
	Inactive	3.24 ± 1.71	3.68 ± 1.18	3.76 ± 0.88	3.39 ± 1.48	1.113	0.345	0.014
Information	Active	3.21 ± 1.50	3.79 ± 1.16	3.87 ± 1.25	3.71 ± 1.55	1.364	0.255	0.017
	Inactive	3.15 ± 1.72	3.57 ± 1.24	3.76 ± 0.94	3.37 ± 1.57	1.217	0.304	0.015
Usability	Active	2.62 ± 1.37	3.17 ± 1.00	3.25 ± 1.03	3.00 ± 1.31	1.725	0.163	0.022
	Inactive	2.54 ± 1.43	2.89 ± 1.01	3.03 ± 0.72	2.72 ± 1.28	1.137	0.335	0.014
Perceived impact	Active	2.81 ± 1.52	3.17 ± 1.23	3.09 ± 1.26	2.65 ± 1.40	0.8221	0.484	0.011
	Inactive	2.66 ± 1.56	2.77 ± 1.14	3.06 ± 0.90	2.75 ± 1.38	0.625	0.599	0.008

**Table 5:** Differences between pre- and post-tests of problematic mobile phone use in active and inactive adolescents.

	Descriptors (M ± SD)								Covariate Quality of the App		
		Mobile App	Pre	Post	Mean Diff.	*p*	95% CI	Effect Size	*p*	95% CI	Effect Size
CERM score	Active	Pokémon Go	16.27 ± 5.30	15.77 ± 7.69	0.500 ± 0.994	0.616	−1.459; 2.459	0.001	0.765	−1.606; 2.182	0.001
		Strava	15.03 ± 3.73	14.45 ± 3.48	0.581 ± 0.978	0.553	−1.347; 2.508	0.002	0.442	−1.134; 2.590	0.003
		Pacer	14.74 ± 4.08	14.65 ± 2.87	0.087 ± 1.136	0.939	−2.151; 2.325	0.001	0.747	−1.809; 2.519	0.001
		MapMyWalk	14.78 ± 2.96	15.56 ± 6.35	−0.778 ± 1.284	0.545	−3.307; 1.752	0.002	0.551	−3.183; 1.702	0.002
	Inactive	Pokémon Go	14.93 ± 5.83	15.31 ± 5.25	−0.379 ± 1.011	0.708	−2.372; 1.614	0.001	0.468	−2.642; 1.219	0.002
		Strava	15.42 ± 3.67	14.86 ± 3.01	0.558 ± 0.831	0.502	−1.078; 2.196	0.002	0.442	−0.963; 2.197	0.003
		Pacer	15.59 ± 5.42	17.41 ± 4.95	−1.811 ± 0.895	0.044	−3.575;−0.047	0.017	0.070	−3.284; 0.130	0.014
		MapMyWalk	14.69 ± 3.49	14.52 ± 5.58	0.172 ± 1.011	0.865	−1.820; 2.165	0.001	0.955	−1.982; 1.872	0.001
Conflictive use	Active	Pokémon Go	6.70 ± 2.52	6.77 ± 3.75	−0.067 ± 0.452	0.883	−0.957; 0.823	0.001	0.738	−1.018; 0.722	0.001
		Strava	6.32 ± 1.78	6.23 ± 1.82	0.097 ± 0.444	0.828	−0.779; 0.972	0.001	0.724	−0.702; 1.009	0.001
		Pacer	6.09 ± 1.70	5.91 ± 0.10	0.174 ± 0.516	0.736	−0.843; 1.191	0.001	0.584	−0.717; 1.271	0.001
		MapMyWalk	6.50 ± 1.54	7.33 ± 4.28	−0.833 ± 0.583	0.154	−1.982; 0.316	0.009	0.152	−1.941 ± 0.303	0.009
	Inactive	Pokémon Go	6.45 ± 2.73	6.69 ± 2.77	−0.241 ± 0.460	0.600	−1.147; 0.664	0.001	0.413	1.256; 0.518	0.003
		Strava	6.28 ± 1.58	6.16 ± 1.54	0.116 ± 0.377	0.758	−0.627; 0.860	0.001	0.707	−0.587; 0.865	0.001
		Pacer	6.43 ± 2.18	7.22 ± 2.44	−0.784 ± 0.407	0.055	−1.585; 0.018	0.016	0.082	−1.478; 0.090	0.013
		MapMyWalk	5.93 ± 1.69	6.24 ± 2.55	−0.310 ± 0.460	0.500	−1.216; 0.595	0.002	0.377	−1.283; 0.488	0.003
Emotional use	Active	Pokémon Go	9.57 ± 3.29	9.00 ± 4.30	0.567 ± 0.621	0.362	−0.656; 1.790	0.004	0.469	−0.748; 1.620	0.002
		Strava	8.71 ± 2.47	8.23 ± 2.25	0.484 ± 0.611	0.429	−0.719; 1.687	0.003	0.332	−0.589; 1.738	0.004
		Pacer	8.65 ± 2.71	8.74 ± 2.36	−0.087 ± 0.709	0.902	−1.484; 1.310	0.001	0.909	−1.274; 1.431	0.001
		MapMyWalk	8.28 ± 1.78	8.22 ± 2.53	0.056 ± 0.801	0.945	−1.523; 1.635	0.001	0.919	−1.448; 1.605	0.001
	Inactive	Pokémon Go	8.48 ± 3.47	8.62 ± 2.90	−0.138 ± 0.631	0.827	−1.382; 1.106	0.001	0.576	−1.549; 0.863	0.001
		Strava	9.14 ± 2.51	8.70 ± 1.95	0.442 ± 0.519	0.395	−0.580; 1.463	0.003	0.341	−0.509; 1.466	0.004
		Pacer	9.16 ± 3.68	10.19 ± 2.94	−1.027 ± 0.559	0.067	−2.128; 0.074	0.014	0.104	−1.950; 0.184	0.011
		MapMyWalk	8.76 ± 2.33	8.28 ± 3.38	0.483 ± 0.631	0.445	−0.761; 1.727	0.003	0.575	−0.861; 1.547	0.001

**Table 6:** Correlation analysis between training volume, app rating and the overall level of problematic mobile phone use in active and inactive adolescents.

**Inactive**									
	**Training Volume**	**Engagement**	**Functionality**	**Aesthetics**	**Information**	**Usability**	**Perceived Impact**	**CERM Total Score**	**Conflictive Use**
Training volume	-	-	-	-	-	-	-		-
Engagement	0.007; *p* = 0.933	-	-	-	-	-	-		-
Functionality	0.034; *p* = 0.695	0.905; *p* < 0.001	-	-	-	-	-		-
Aesthetics	−0.001; *p* = 0.992	0.870; *p* < 0.001	0.926; *p* < 0.001	-	-	-	-		-
Information	0.074; *p* = 0.387	0.859; *p* < 0.001	0.911; *p* < 0.001	0.927; *p* < 0.001	-	-	-		-
Usability	−0.013; *p* = 0.884	0.804; *p* < 0.001	0.812; *p* < 0.001	0.822; *p* < 0.001	0.824; *p* < 0.001	-	-		-
Perceived impact	−0.067; *p* = 0.432	0.732; *p* < 0.001	0.777; *p* < 0.001	0.759; *p* < 0.001	0.740; *p* < 0.001	0.827; *p* < 0.001	-		-
CERM total score	−0.114; *p* = 0.184	0.106; *p* = 0.218	0.125; *p* = 0.144	0.091; *p* = 0.290	0.099; *p* = 0.250	0.158; *p* = 0.064	0.176; *p* = 0.038		-
Conflictive use	−0.118; *p* = 0.168	0.077; *p* = 0.369	0.082; *p* = 0.340	0.079; *p* = 0.360	0.088; *p* = 0.304	0.162; *p* = 0.058	0.198; *p* = 0.020	0.902; *p* < 0.001	
Emotional use	−0.094; *p* = 0.274	0.114; *p* = 0.184	0.142; *p* = 0.096	0.088; *p* = 0.307	0.093; *p* = 0.278	0.132; *p* = 0.122	0.133; *p* = 0.119	0.936; *p* < 0.001	0.693; *p* < 0.001
**Active**									
	**Training volume**	**Engagement**	**Functionality**	**Aesthetics**	**Information**	**Usability**	**Perceived Impact**	**CERM Total Score**	**Conflictive Use**
Training volume	-	-	-	-	-	-	-		-
Engagement	0.080; *p* = 0.423	-	-	-	-	-	-		-
Functionality	0.015; *p* = 0.879	0.884; *p* < 0.001	-	-	-	-	-		-
Aesthetics	−0.070; *p* = 0.485	0.878; *p* < 0.001	0.907; *p* < 0.001	-	-	-	-		-
Information	−0.009; *p* = 0.927	0.833; *p* < 0.001	0.868; *p* < 0.001	0.876; *p* < 0.001	-	-	-		-
Usability	−0.006; *p* = 0.952	0.820; *p* < 0.001	0.853; *p* < 0.001	0.834; *p* < 0.001	0.896; *p* < 0.001	-	-		-
Perceived impact	−0.056; *p* = 0.573	0.681; *p* < 0.001	0.723; *p* < 0.001	0.722; *p* < 0.001	0.791; *p* < 0.001	0.793; *p* < 0.001	-		-
CERM total score	0.225; *p* = 0.023	0.424; *p* < 0.001	0.348; *p* < 0.001	0.311; *p* = 0.001	0.302; *p* = 0.002	0.332; *p* < 0.001	0.190; *p* = 0.055		-
Conflictive use	0.236; *p* = 0.017	0.342; *p* < 0.001	0.264; *p* = 0.007	0.215; *p* = 0.030	0.225; *p* = 0.023	0.256; *p* = 0.009	0.160; *p* = 0.107	0.907; *p* < 0.001	
Emotional use	0.174; *p* = 0.080	0.428; *p* < 0.001	0.368; *p* < 0.001	0.349; *p* < 0.001	0.323; *p* < 0.001	0.347; *p* < 0.001	0.186; *p* = 0.061	0.914; *p* < 0.001	0.659; *p* < 0.001

## Data Availability

The database corresponding to the article is hosted in the open repository “Zenodo” at the following link: https://doi.org/10.5281/zenodo.15070655.

## Abbreviations

The following abbreviations are used in this manuscript:

WHO	World Health Organization
H1	Hypothesis 1
H2	Hypothesis 2
H3	Hypothesis 3
H4	Hypothesis 4
ESO	Compulsory Secondary Education
SD	standard deviation
CI	confidence interval
PAQ-A	Physical Activity Questionnaire for Adolescents questionnaire
CERM	Questionnaire of Mobile-Phone-Related Experiences
uMars	user version mobile application rating scale
χ2	Chi-square test
ES	effect size
